# Utahmycin C, a new glycosylated derivative of a rare azaanthraquinone produced by *Streptomyces* sp. YD248

**DOI:** 10.3389/fmicb.2026.1921234

**Published:** 2026-09-16

**Authors:** Fatemeh M. Tajabadi, Andrew M. Piggott, Jeff Errington, Yousef Dashti

**Affiliations:** 1 School of Medical Sciences, Faculty of Medicine and Health, University of Sydney, Sydney, NSW, Australia; 2 School of Natural Sciences, Macquarie University, Sydney, NSW, Australia; 3 Sydney Infectious Diseases Institute, University of Sydney, Sydney, NSW, Australia

**Keywords:** azaanthraquinone, polyketide, glycoside, streptomyces, type II PKS

## Abstract

Chemical investigation of *Streptomyces* sp. YD248 led to the isolation of utahmycin C (**1**), a new glycosylated member of the rare bacterial 1,3-dimethyl-2-azaanthraquinone family. Its structure was established by NMR spectroscopy and high-resolution mass spectrometry, revealing a *β*-D-glucuronic acid substituent attached to the azaanthraquinone core. Unusual long-range *^5^J* HMBC correlations prompted validation by density functional theory (DFT) through NMR chemical shift calculations, which showed excellent agreement with the experimental data. Bioinformatics analysis identified a biosynthetic gene cluster (BGC) homologous to the reported Erd gene cluster associated with utahmycin A biosynthesis. This study expands the structural diversity of bacterial azaanthraquinones.

## Introduction

Azaanthraquinones constitute a rare class of natural products that exhibit a broad spectrum of biological activities, including antimicrobial and potential anticancer effects (Koyama et al., 2005; Okunade et al., 1999; Nok, 2002). To date, naturally occurring azaanthraquinones have been isolated predominantly from lichens and fungi, where they typically occur as derivatives of 3-methyl-2-azaanthraquinones (Figure 1) (Nok, 2002; Arsenault, 1965; Miljkovic et al., 2001; Moriyasu et al., 2001; Parisot et al., 1989; Gräfe et al., 1990). Their occurrence in bacteria is considerably rarer, and all bacterial examples reported to date possess a 1,3-dimethyl-2-azaanthraquinone scaffold (Bauer et al., 2010; Bunbamrung et al., 2018; Abdelfattah et al., 2012). Utahmycin A (**2**) was the first reported example of this class produced by bacteria (Bauer et al., 2010). Two additional bacterial metabolites possessing closely related 2-azaanthraquinone-derived scaffolds have subsequently been reported. Azanthromicin A (**3**) is a reduced derivative of the 1,3-dimethyl-2-azaanthraquinone core with additional methyl substituents at C-6 and C-10 (Bunbamrung et al., 2018), while katorazone (**4**) appears to arise from condensation of the carbonyl group of utahmycin A with methyl 2-hydrazinylbenzoate (Abdelfattah et al., 2012). Utahmycin A was initially identified following heterologous expression of an environmental DNA-derived type II polyketide synthase (PKS) gene cluster in *S. albus* (Bauer et al., 2010), and was later detected in culture extracts of *Streptomyces* sp. IFM 11299 together with katorazone **4** (Abdelfattah et al., 2012).

**Figure 1:**
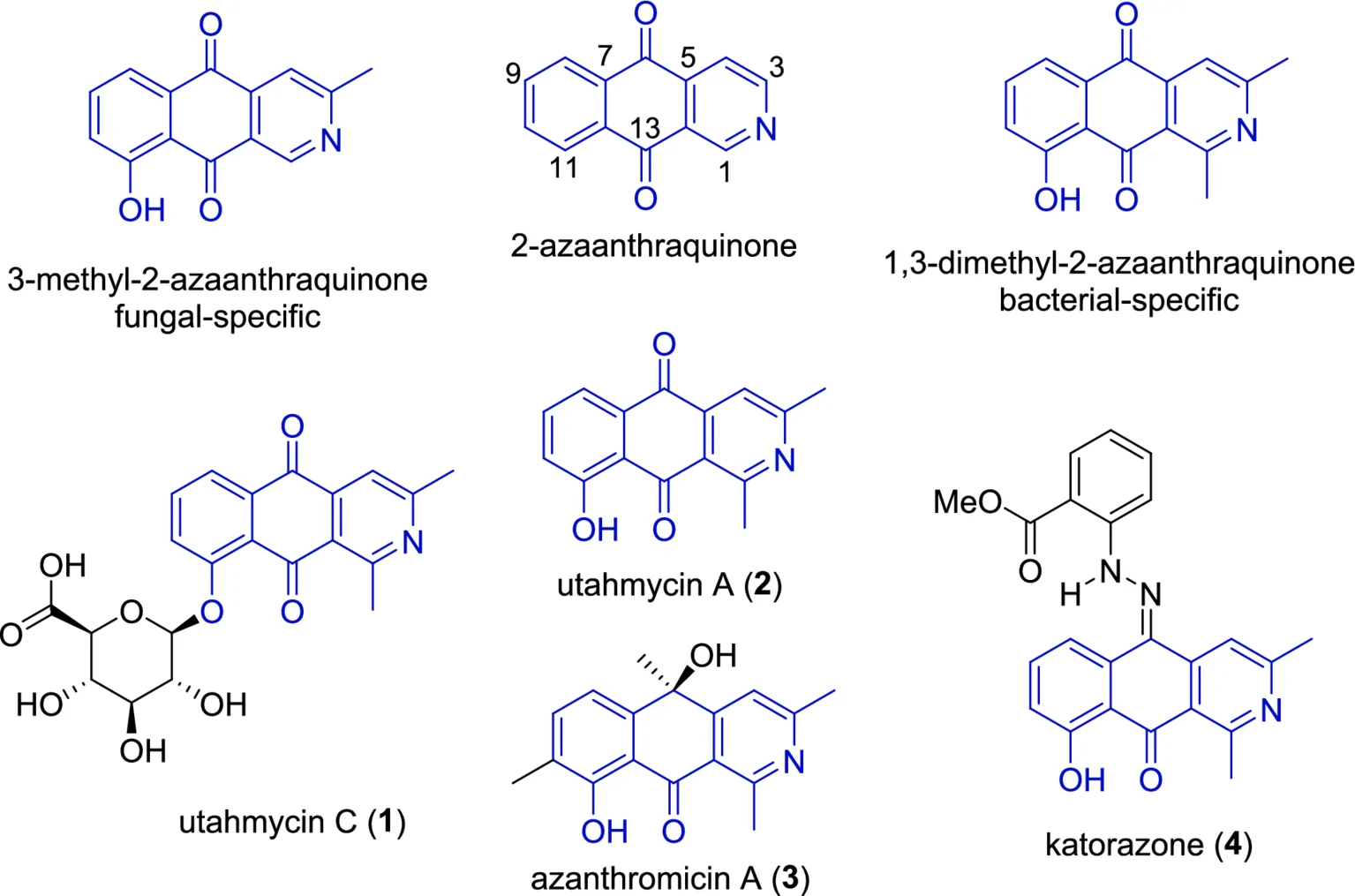
Core scaffolds of 2-azaanthraquinone, fungal-specific 3-methyl-2-azaanthraquinone, and bacterial-specific 1,3-dimethyl-2-azaanthraquinone. Structures of all bacterially derived 2-azaanthraquinones reported to date (**2**–**4**) and the new analogue Utahmycin C (**1**) characterized in this study.

During our ongoing investigation of specialized metabolites produced by actinomycetes, we identified a previously undescribed glycosylated 1,3-dimethyl-2-azaanthraquinone, designated utahmycin C (**1**) (Figure 1) from *Streptomyces* sp. YD248. Herein, we report the isolation, structure elucidation, and computational verification of utahmycin C (**1**).

## Results and discussion

In a screening of a microbial extract library against *Candida albicans*, the organic extract of a culture of *Streptomyces* strain YD248 showed potent antifungal activity. Mass spectrometry-based dereplication indicated the production of known antifungal compounds, including candicidins A1-A3 (also known as ascosins A1-A3 and levorins A1-A3) (Szczeblewski et al., 2017), as well as the siderophores desferrioxamine B and E (Supplementary Figures S1, S2). In addition, an ion at *m*/*z* 430.1135 [M + H]^+^ (calcd for C_21_H_20_NO_9_^+^, 430.1133) corresponded to an unknown metabolite. To elucidate its structure, the compound was isolated from a 1 L GYM liquid culture. Compound **1** was obtained as a white amorphous solid following MS-guided fractionation using preparative HPLC.

Analysis of the NMR spectra of compound **1** (Figure 2, Table 1, and Supplementary Figures S3–S7) revealed signals corresponding to an extensively conjugated aromatic system, together with additional resonances consistent with a carbohydrate-derived unit. The ^13^C NMR spectrum showed 15 resonances attributable to the aromatic core, including two carbonyl carbons at *δ_C_* 183.0 and 183.1, oxygenated or heteroatom-substituted aromatic carbons at *δ_C_* 156.6, 159.3, and 162.7, and two methyl carbons at *δ_C_* 25.4 and 24.5. Detailed analysis of the 1D and 2D NMR data established a 1,3-dimethyl-2-azaanthraquinone framework, as supported by key HMBC correlations (Figure 2).

**Figure 2:**
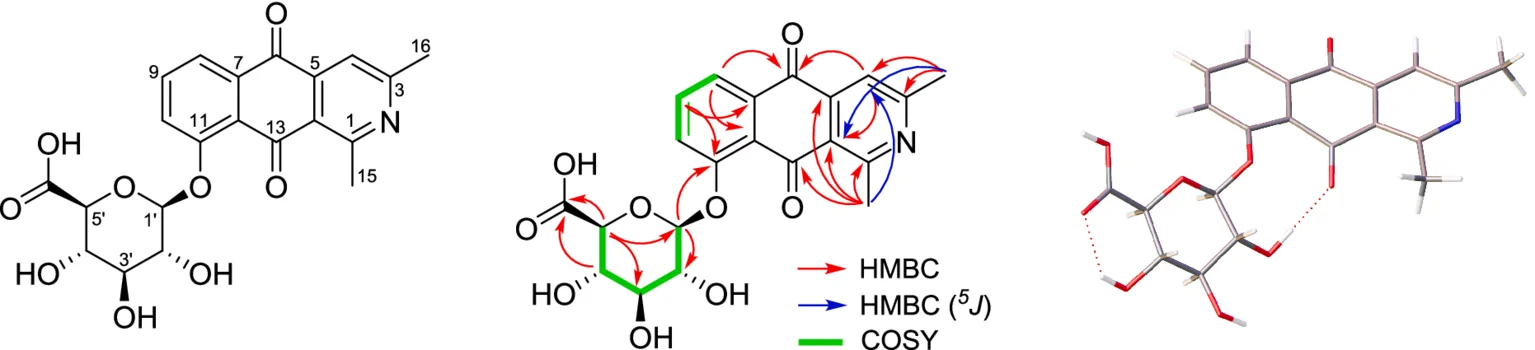
Structure of utahmycin C (**1**) with numbering used in NMR Table 1 (left), key HMBC correlations (red arrows), including unusual *^5^J* correlations (blue arrows), and the energy-minimized lowest energy conformer (right).

**Table 1:** ^1^H/^13^C NMR chemical shifts and HMBC correlations of utahmycin C (1) in DMSO-*d*_6_. DFT-calculated ^13^C NMR chemical shifts are shown for comparison.

Position	Experimental			Calculated	
	*δ_C_*	*δ_H_*, mult (*J* in Hz)	HMBC correlations	*δ_C_*	Error (Δ*δ_C_*)
1	159.3			160.0	−0.7
3	162.7			164.2	−1.5
4	116.1	7.72, s	C3, C6, C14, C16	113.6	2.5
5	139.2			136.4	2.8
6	183.0			182.6	0.4
7	134.0			130.8	3.2
8	120.2	7.82, *ol*	C6, C10, C12	120.8	−0.6
9	134.7	7.82, *ol*	C7, C11	133.8	0.9
10	122.9	7.69, m	C8, C9, C11, C12	124.6	−1.7
11	156.6			156.8	−0.2
12	123.4			122.5	0.9
13	183.1			185.3	−2.2
14	124.4			121.3	3.1
15	25.4	2.87, s	C1, C4, C5, C13, C14	27.5	−2.1
16	24.5	2.61, s	C3, C4, C6, C14	24.9	−0.4
1′	100.5	5.27, d (7.6)	C11, C2′	104.6	−4.1
2′	73.1	3.48, t (8.3)	C3′	74.1	−1.0
3′	75.7	3.35, t (9.0)	C1′, C2′, C4′	75.1	0.6
4′	71.3	3.45, t (9.5)	C5′, C6′	71,8	−0.5
5′	75.5	3.98, d (9.7)	C1′, C3′, C4′, C6′	73.2	2.3
6′	170.1			171.8	−1.7

The carbohydrate-derived unit was characterised based on its characteristic ^1^H and ^13^C NMR resonances. The proton spin system spanning CH-1′ to CH-5′ was established from 2D NMR data (COSY and HSQC). This spin system was connected to the carboxyl carbon resonance at *δ_C_* 170.1 (C-6′) via HMBC correlations from H-4′ and H-5′. The carboxyl carbon resonance of C-6′ is consistent with oxidation of the C-6 hydroxymethyl group, supporting assignment as a glucuronic acid residue. The relatively large coupling constant of the anomeric proton at *δ_H_* 5.27 (d, *J* = 7.6 Hz) indicated a trans-diaxial relationship between H-1′ and H-2′, consistent with a *β*-configuration. Further support for this assignment was provided by the large vicinal coupling constants observed throughout the sugar spin system [H-2′, *δ_H_* 3.48 (t, *J* = 8.3 Hz); H-3′, *δ_H_* 3.35 (t, *J* = 9.0 Hz); H-4′, *δ_H_* 3.45 (t, *J* = 9.5 Hz); H-5′, *δ_H_* 3.98 (d, *J* = 9.7 Hz)], consistent with a pyranose ring adopting a ^4^C₁ chair conformation with predominantly axial proton orientations. These data are in agreement with a *β*-D-glucuronic acid residue. The sugar attachment site on the aglycone was established by an HMBC correlation from the anomeric proton H-1′ (*δ_H_* 5.27) to the oxygenated aromatic carbon C-11 (*δ_C_* 156.6), locating the *β*-D-glucuronyl substituent at C-11 of the azaanthraquinone core.

The aromatic methyl groups CH_3_-15 and CH_3_-16 of the 1,3-dimethyl-2-azaanthraquinone aglycone exhibited weak but unusual *^5^J* HMBC correlations to C-4 and to C-14, respectively. Although such long-range correlations are uncommon, they can occasionally be observed in HMBC experiments optimised for detecting *^2^J* and *^3^J* couplings, particularly in rigid aromatic systems, where extended π-conjugation can facilitate efficient magnetisation transfer (Araya-Maturana et al., 2008). Examples of compounds in which long-range *^4^J* or *^5^J* HMBC correlations have been reported include purine and carbazolequinone derivatives (Procházková et al., 2012; Marek and Sklenář, 2004; Monroy-Cárdenas et al., 2023). Although H-4 (*δ_H_* 7.72) exhibited an HMBC correlation only to C-16, with no corresponding correlation to C-15, consistent with the proposed structure, the unexpected *^5^J* correlations nevertheless prompted further computational analysis to provide additional support for the structural assignment.

To validate the proposed structure of **1**, DFT calculations were performed. The initial conformational analysis identified the lowest-energy conformer, which was subsequently subjected to geometry optimisation at the B3LYP-D3(BJ)/def-TZVP level of theory (Supplementary Table S1). Solvent effects were incorporated using the COSMO solvation model with the dielectric constant of DMSO to mimic the experimental NMR conditions. Following geometry optimisation, NMR shielding tensors were calculated using the gauge-independent atomic orbitals (GIAO) method at the same level of theory, and linear regression was used to calculate ^13^C chemical shifts from the isotropic shielding constants (Supplementary Figure S8). The calculated ^13^C chemical shifts showed excellent agreement with the experimental values (Table 1, and Supplementary Figure S8), with an RMSD of 1.9 ppm. In particular, the chemical shifts of carbons surrounding the methyl substituents and the glycosylation site were reproduced accurately, supporting the proposed substitution pattern of the aromatic nucleus. The computational results provide strong evidence for the assigned structure and support interpretation of the unusual HMBC correlations involving the aromatic methyl groups as genuine long-range coupling phenomena within the highly conjugated azaanthraquinone framework.

Compound **1** was evaluated for antibacterial activity against *Escherichia coli*, *Staphylococcus aureus*, and *Bacillus subtilis*, and antifungal activity against *Candida albicans* using a previously described method (Sumang et al., 2025). At concentrations up to 64 μg/mL it did not display significant antibacterial or antifungal activity against the tested microorganisms.

The structural differences between fungal 3-methyl-2-azaanthraquinones and bacterial 1,3-dimethyl-2-azaanthraquinones have been rationalized by distinct polyketide biosynthetic folding paradigms and differences in the lengths of their proposed polyketide precursors. Fungal 2-azaanthraquinones originate from the fungal-specific (“F-type”) cyclization of a heptaketide precursor (Thomas, 2001; Van Wagoner et al., 2008), whereas bacterial 1,3-dimethyl-2-azaanthraquinones are proposed to arise from the *Streptomyces*-specific (“S-type”) folding of a decarboxylated octaketide intermediate (Bauer et al., 2010; King et al., 2009). These contrasting biosynthetic pathways account for the characteristic substitution patterns observed in fungal and bacterial azaanthraquinones. The biosynthesis of utahmycin A has been proposed to be directed by the Erd BGC encoding a type II PKS (Bauer et al., 2010). Bioinformatic analysis (Blin et al., 2025) of the genome sequence of *Streptomyces* strain YD248 identified a homologous Erd BGC (Supplementary Figure S9), suggesting that **1** is biosynthesised via the same core pathway as **2**, followed by glycosylation of the aglycone (Figure 3). Notably, the Erd homologue identified in strain YD248 lacks genes encoding glycosyltransferases and other enzymes typically involved in sugar biosynthesis and tailoring, indicating that glycosylation of **2** to yield **1** is likely mediated by enzymes encoded elsewhere in the genome. In addition to the Erd homologue, the genome of *Streptomyces* strain YD248 contains biosynthetic gene clusters for candicidins and desferrioxamines (Supplementary Figure S9), consistent with the MS-based identification of these metabolites.

**Figure 3:**
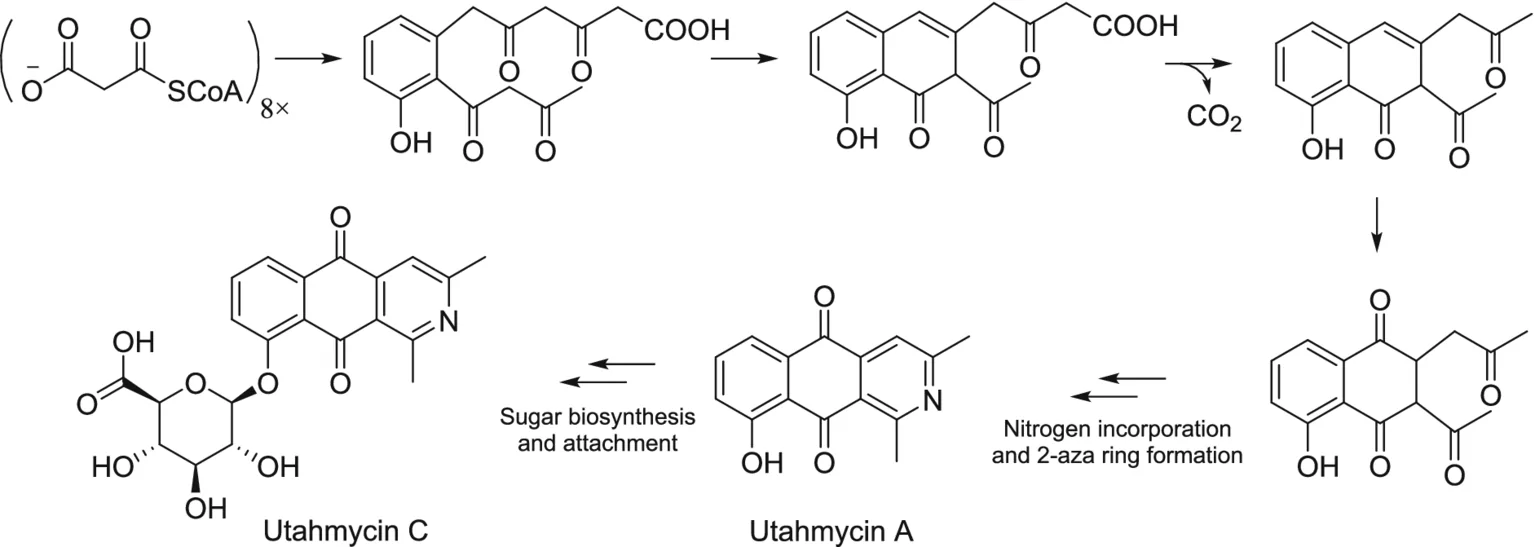
Proposed biosynthesis of Utahmycin C (**1**) by a type II polyketide synthase pathway.

## Experimental section

**General experimental procedures:** NMR spectra were recorded on a Bruker Avance III 700 MHz spectrometer equipped with a TCI cryoprobe at 25 °C. The ^1^H and ^13^C NMR chemical shifts were referenced to the solvent peak of DMSO-*d*_6_ at *δ*_H_ 2.50 and *δ*_C_ 39.51. LC–HRMS analysis was performed on a Thermo Vanquish UHPLC system equipped with a Poroshell 120 EC-C18 column (2.1 × 100 mm, 1.9 μm) and coupled to a Thermo Orbitrap 120 Exploris mass spectrometer. Preparative HPLC purification was carried out using an Agilent 1260 Infinity II system fitted with a semi-preparative reverse phase C_18_ Betasil column, 5 μm (21.2 × 150 mm). All LC–MS and HPLC separations were performed using acetonitrile (MeCN)–H_2_O gradient systems. HPLC grade solvents and Milli-Q water (Millipore) were used throughout.

**Production, extraction and HPLC purification:** *Streptomyces* sp. YD248, isolated from a plant rhizosphere soil sample, was cultured in 1 L of GYM medium (Glucose. 4.0 g/L. Yeast extract. 4.0 g/L. Malt extract. 10.0 g/L) at 30 °C for 7 days. Following centrifugation and filtration to remove bacterial biomass, the culture supernatant was extracted with ethyl acetate (2 × 1 L). The resulting organic extract was concentrated under reduced pressure and redissolved in 800 μL DMSO for purification by preparative HPLC. Fractionation was performed using an isocratic elution of 5% MeCN for 5 min, followed by a linear gradient to 100% MeCN over 45 min and a final isocratic hold at 100% MeCN for 10 min. Fractions were collected at 30 s intervals, yielding pure metabolite 1 in fraction 56.

*Utahmycin C* (**1**): white solid (5 mg/L); ^1^H NMR (700 MHz, DMSO-*d*_6_) and ^13^C NMR (175 MHz, DMSO-*d*_6_), see Table 1; HRESIMS *m*/*z* 430.1135 [M + H]^+^ (calcd for C_21_H_20_NO_9_^+^, 430.1133).

**DFT calculations:** Conformers of **1** were generated using Confab (Open Babel v3.1.1) (O’Boyle et al., 2011a; O’Boyle et al., 2011b), and relative energies were calculated using the MMFF94 force field. The lowest-energy conformer was geometry-optimised by DFT (B3LYP-D3(BJ)/def-TZVP) with COSMO solvation (DMSO), and NMR shielding tensors were calculated using the GIAO method at the same level of theory (Turbomole v7.5) (TURBOMOLE, 2020).

**Genomic DNA extraction, genome sequencing, assembly, and error correction:** *Streptomyces* strain YD248 was inoculated in 10 mL liquid GYM culture medium and incubated for 3 days at 30 °C and 120 rpm. 300 μL of the cell culture was used for genomic DNA extraction using the Quick-DNA HMW MagBead Kit (Zymo Research, cat. no. D6060). DNA quality was assessed by agarose gel electrophoresis to ensure the absence of RNA. DNA concentration was assessed using the dsDNA assay on a Qubit fluorometer. *Streptomyces* strain YD248 was a part of a multiplexed nanopore MinION sequencing run (12 strains in total) using the Native Barcoding Expansion 1–12 (EXP-NDB104) and Ligation Sequencing Kit (SQK-LSK109). DNA library preparation, barcoding, and adapter ligation was performed according to the manufacturer’s instructions. The MinION long reads were assembled using Flye (version 2.8.1) (Kolmogorov et al., 2019) and error-corrected using minimap2 (version 2.17) (Li, 2018), Racon (version 1.4.13) (Vaser et al., 2017) and medaka (version 1.0.3) (Medaka, 2018).

## Conclusion

A new glycosylated azaanthraquinone derivative, utahmycin C (**1**), was isolated from a *Streptomyces* strain and structurally characterized by spectroscopic methods. DFT calculations provided strong support for the proposed structure and confirmed the substitution pattern of the aromatic core. Utahmycin C represents the first glycosylated member of the bacterial 1,3-dimethyl-2-azaanthraquinone family, further expanding the limited structural diversity currently known for this rare class of microbial natural products.

## Data Availability

The data analyzed in this study are available in Figshare at 10.6084/m9.figshare.33474313.
