# Supplementary material for: Utahmycin C, a new glycosylated derivative of a rare azaanthraquinone produced by Streptomyces sp. YD248
Source: Front Microbiol. 2026 Sep 16;17:1921234. doi: 10.3389/fmicb.2026.1921234 (PMC13625050; doi:10.3389/fmicb.2026.1921234)
Supplement: Supplementary file 1 [file Supplementary_file_1.DOCX]

**Supplementary Information**

**Utahmycin C, a new glycosylated derivative of a rare azaanthraquinone produced by *Streptomyces* sp. YD248**

Fatemeh M. Tajabadi,^a^ Andrew M. Piggott,^b^ Jeff Errington,^ac^ Yousef Dashti^ac*^

^a^ Faculty of Medicine and Health, University of Sydney, Sydney NSW 2006, Australia

^b^ School of Natural Sciences, Macquarie University, Sydney, NSW 2109, Australia

^c^ Sydney Infectious Diseases Institute, University of Sydney, Sydney NSW 2006, Australia

^*^ Corresponding author: [yousef.dashti@sydney.edu.au](mailto:yousef.dashti@sydney.edu.au)

| **contents** | **Page** |
| --- | --- |
| Figure S1. HR-MS spectra of candicidins/ ascosins/ levorins A1-A3. | S2 |
| Figure S2. HR-MS spectra of desferrioxamine B and E. | S3 |
| Figure S3. ^1^H NMR spectrum of utahmycin C (**1**) recorded at 700 MHz in DMSO-*d*_6_. | S4 |
| Figure S4. ^13^C NMR spectrum of utahmycin C (**1**) recorded at 175 MHz in DMSO-*d*_6_. | S5 |
| Figure S5. COSY spectrum of utahmycin C (**1**) recorded at 700 MHz in DMSO-*d*_6_. | S6 |
| Figure S6. HSQC spectrum of utahmycin C (**1**) recorded at 700 MHz in DMSO-*d*_6_. | S7 |
| Figure S7. HMBC spectrum of utahmycin C (**1**) recorded at 700 MHz in DMSO-*d*_6_. | S8 |
| Table S1. XYZ coordinates for DFT-optimised structure of utahmycin C (**1**). | S9 |
| Figure S8. DFT-calculated isotropic shielding constants vs. experimental ^13^C NMR chemical shifts for utahmycin C (**1**). | S10 |
| Table S2. ^1^H/^13^C NMR chemical shifts of utahmycin C (**1**) in DMSO-*d*_6_ (700/175 MHz) compared to reported values for utahmycin A (**2**) in CD_2_Cl_2_-*d*_6_ (600/200 MHz). | S11 |
| Figure S9. AntiSMASH output of the genome sequence of *Streptomyces* strain YD248. | S12 |

**Figure S1.** HR-MS spectra of candicidins/ascosins/levorins A1-A3.

**Figure S2.** HR-MS spectrum of desferrioxamine B and E.

**Figure S3.** ^1^H NMR spectrum of utahmycin C (**1**) recorded at 700 MHz in DMSO-*d*_6_.

**Figure S4.** ^13^C NMR spectrum of utahmycin C (**1**) recorded at 175 MHz in DMSO-*d*_6_.

**Figure S5.** COSY spectrum of utahmycin C (**1**) recorded at 700 MHz in DMSO-*d*_6_.

**Figure S6.** HSQC spectrum of utahmycin C (**1**) recorded at 700 MHz in DMSO-*d*_6_.

**Figure S7.** HMBC spectrum of utahmycin C (**1**) recorded at 700 MHz in DMSO-*d*_6_.

**Table S1**. XYZ coordinates for DFT-optimised structure of utahmycin C (**1**).

| C | 3.392452 | -4.609003 | 0.914737 |
| --- | --- | --- | --- |
| C | 3.230612 | -3.285165 | 1.648477 |
| C | 1.796444 | -2.781470 | 1.559210 |
| C | 1.340681 | -2.752249 | 0.108366 |
| O | 1.440141 | -4.045047 | -0.467040 |
| C | 2.789192 | -4.490784 | -0.503294 |
| O | 1.747821 | -1.484012 | 2.127598 |
| O | 3.618930 | -3.474707 | 3.004837 |
| O | 4.778734 | -4.905949 | 0.849361 |
| C | 2.846528 | -5.816780 | -1.244912 |
| O | 3.699526 | -6.654342 | -1.030315 |
| O | -0.012604 | -2.381300 | 0.120835 |
| O | 1.901705 | -5.946682 | -2.169034 |
| C | -0.750713 | -1.180379 | -3.259978 |
| C | -0.195737 | -1.927050 | -2.229838 |
| C | -0.495781 | -1.617048 | -0.905108 |
| C | -1.359158 | -0.556724 | -0.589147 |
| C | -1.950902 | 0.151791 | -1.654345 |
| C | -1.635228 | -0.148369 | -2.976049 |
| C | -1.597128 | -0.112374 | 0.813224 |
| C | -2.817063 | 0.686460 | 1.071133 |
| C | -3.444682 | 1.354663 | 0.011566 |
| C | -2.939778 | 1.223386 | -1.386273 |
| C | -3.362262 | 0.839264 | 2.367150 |
| N | -4.441642 | 1.601144 | 2.574292 |
| C | -5.023582 | 2.260403 | 1.561272 |
| C | -4.547810 | 2.155917 | 0.257380 |
| O | -3.362482 | 1.947084 | -2.274969 |
| O | -0.806815 | -0.352790 | 1.720805 |
| C | -2.817793 | 0.155841 | 3.587538 |
| C | -6.213182 | 3.105150 | 1.901083 |
| H | 2.853010 | -5.388863 | 1.462996 |
| H | 3.887753 | -2.547330 | 1.170929 |
| H | 1.137240 | -3.472029 | 2.098047 |
| H | 1.925613 | -2.033654 | -0.477202 |
| H | 3.407650 | -3.789425 | -1.081766 |
| H | 0.840822 | -1.135965 | 2.013199 |
| H | 3.492113 | -2.632638 | 3.463106 |
| H | 4.858401 | -5.751328 | 0.379971 |
| H | 2.017738 | -6.801159 | -2.621694 |
| H | -0.510337 | -1.423791 | -4.286605 |
| H | 0.453508 | -2.765236 | -2.444020 |
| H | -2.095554 | 0.422165 | -3.770306 |
| H | -5.026098 | 2.682725 | -0.555882 |
| H | -2.585597 | -0.891622 | 3.396820 |
| H | -1.888492 | 0.628138 | 3.911047 |
| H | -3.553072 | 0.233311 | 4.386182 |
| H | -6.998514 | 2.486024 | 2.341149 |
| H | -5.938133 | 3.857325 | 2.643914 |
| H | -6.611374 | 3.606672 | 1.020351 |

| DFT-Calculated Isotropic Shielding Constant | Experimental Chemical Shift (δC) |
| --- | --- |
| 13.6825 | 159.3 |
| 9.10236 | 162.7 |
| 63.3905 | 116.1 |
| 38.9829 | 139.2 |
| -10.5948 | 183 |
| 44.8951 | 134 |
| 55.6725 | 120.2 |
| 41.6914 | 134.7 |
| 51.5536 | 122.9 |
| 17.0606 | 156.6 |
| 53.8217 | 123.4 |
| -13.4708 | 183.1 |
| 55.1741 | 124.4 |
| 155.743 | 25.4 |
| 158.527 | 24.5 |
| 73.0097 | 100.5 |
| 105.793 | 73.1 |
| 104.639 | 75.7 |
| 108.209 | 71.3 |
| 106.76 | 75.5 |
| 0.977224 | 170.1 |

| Carbon Atom Number | Error in Chemical Shift (ΔδC) |
| --- | --- |
| 1 | -0.652417 |
| 3 | -1.52296 |
| 4 | 2.49534 |
| 5 | 2.83768 |
| 6 | 0.411453 |
| 7 | 3.15019 |
| 8 | -0.601004 |
| 9 | 0.863021 |
| 10 | -1.74146 |
| 11 | -0.202714 |
| 12 | 0.873362 |
| 13 | -2.17016 |
| 14 | 3.13433 |
| 15 | -2.09499 |
| 16 | -0.399309 |
| 1' | -4.13576 |
| 2' | -0.969031 |
| 3' | 0.55577 |
| 4' | -0.515484 |
| 5' | 2.33261 |
| 6' | -1.69884 |

**Figure S8**. DFT-calculated isotropic shielding constants vs. experimental ^13^C NMR chemical shifts for utahmycin C (**1**).

**Table S2.** ^1^H/^13^C NMR chemical shift of utahmycin C (**1**) in DMSO-*d*_6_ (700/175 MHz) compared to reported values for utahmycin A (**2**) in CD_2_Cl_2_ (600/200 MHz).

|  | **1** **2** | | | |
| --- | --- | --- | --- | --- |
| Position | *δ_C_* | *δ_H_* | *δ_C_* | *δ_H_* |
| 1 | 159.3 |  | 162.1 |  |
| 3 | 162.7 |  | 165.2 |  |
| 4 | 116.1 | 7.72 | 117.8 | 7.85 |
| 5 | 139.2 |  | 117.9* |  |
| 6 | 183.0 |  | 183.1 |  |
| 7 | 134.0 |  | 133.1 |  |
| 8 | 120.2 | 7.82 | 119.5 | 7.77 |
| 9 | 134.7 | 7.82 | 136.9 | 7.69 |
| 10 | 122.9 | 7.69 | 125.8 | 7.35 |
| 11 | 156.6 |  | 163.1 |  |
| 12 | 123.4 |  | 117.2 |  |
| 13 | 183.1 |  | 190.3 |  |
| 14 | 124.4 |  | 122.8 |  |
| 15 | 25.4 | 2.87 | 26.8 | 3.05 |
| 16 | 24.5 | 2.61 | 25.2 | 2.70 |
| 1′ | 100.5 | 5.27 |  |  |
| 2′ | 73.1 | 3.48 |  |  |
| 3′ | 75.7 | 3.35 |  |  |
| 4′ | 71.3 | 3.45 |  |  |
| 5′ | 75.5 | 3.98 |  |  |
| 6′ | 170.1 |  |  |  |

* The reported C-5 ^13^C NMR chemical shift of utahmycin A appears to be misassigned, as DFT calculations for compound **2** predict a value of approximately 139 ppm.

**Figure S9**. AntiSMASH output of the genome sequence of *Streptomyces* strain YD248.
